# Spontaneous Epidural Hematomas in a Patient With Sickle Cell Anemia: A Case Report

**DOI:** 10.7759/cureus.71257

**Published:** 2024-10-11

**Authors:** Ruby R Taylor, Natally M Santiago, Danny L John, Joacir G Cordeiro, Felipe M Do Rego Monteiro

**Affiliations:** 1 Neurological Surgery, University of Miami Miller School of Medicine, Miami, USA; 2 Medical Scientist Training Program, University of Miami Miller School of Medicine, Miami, USA; 3 Neurological Surgery, Hospital Beneficencia Portuguesa de São Paulo, São Paulo, BRA

**Keywords:** bone infarction, epidural hematoma, hemorrhagic complications, neurosurgery, sickle cell disease

## Abstract

Sickle cell disease (SCD) is a systemic organ disease with acute and chronic complications. Neurological complications of SCD include cerebral ischemia, moyamoya syndrome, posterior reversible encephalopathy syndrome, cerebral fat embolism, and cerebral venous sinus thrombosis. Although less frequent, rare hemorrhagic manifestations, such as spontaneous epidural hematoma (EDH), can occur and are associated with increased mortality and morbidity. Herein, we present a case of a 20-year-old male with SCD who developed a massive, bilateral EDH without a history of trauma. MRI revealed ischemic bone changes associated with the hematoma, suggesting bone infarction as the underlying mechanism. This case highlights the importance of considering hemorrhagic complications in the differential diagnosis of SCD patients with acute neurological symptoms, even in the absence of a recent vaso-occlusive crisis.

## Introduction

Sickle cell disease (SCD) is a hereditary blood disorder resulting from a genetic mutation, primarily affecting individuals of African descent [1]. The disorder is caused by a mutation in the beta-globin gene (HBB), which leads to the production of abnormal hemoglobin [2]. This single amino acid substitution, which substitutes valine for glutamic acid in codon 6 of the beta-globin chain, results in hemoglobin polymerization under low oxygen conditions, causing red blood cells to take on a sickle shape [1,3]. The resulting distorted red blood cells disrupt normal blood flow, leading to hemolysis, ischemia, and vaso-occlusive crises. Patients with SCD frequently present with anemia, fatigue, fever, muscle pain, and progressive organ damage due to micro- and macrovascular occlusions [1-3]. Neurologic manifestations are common in SCD, affecting nearly 35% of individuals and significantly contributing to morbidity and mortality [4]. These are predominantly ischemic in nature, including cerebral ischemia, moyamoya syndrome, posterior reversible encephalopathy syndrome, cerebral fat embolism, and cerebral venous sinus thrombosis [4]. However, while ischemic complications dominate the clinical landscape, the literature on hemorrhagic complications remains sparse. Rarely, spontaneous epidural hematoma (EDH) can occur, and when it does, it is typically associated with coagulopathies, tumors, vascular malformations, or infectious diseases [4-7]. The occurrence of spontaneous EDH in SCD is exceedingly rare, making this case significant. This report aims to present this uncommon hemorrhagic complication in an SCD patient, underscoring the critical need for prompt recognition and treatment.

## Case presentation

A 20-year-old male with a known history of SCD was transferred to Ryder Trauma Center, Miami, USA after presenting with a massive bifrontal EDH. Two days prior, the patient abruptly lost consciousness following a severe headache and was transported to a local hospital by emergency medical services, where he remained for 36 hours before being transferred to our facility. He arrived intubated and exhibited bilateral fixed, non-reactive pupils, absent gag reflex, and, after painful stimulus, a decorticate posturing with no eye-opening (E1, V1, M3 - Glasgow Coma Scale 5), indicating a severe brain injury. The remainder of the physical examination was unremarkable. Vital signs were within normal limits, and laboratory studies revealed anemia with a slightly increased international normalized ratio (INR) but no evidence of coagulopathy (Table 1). The patient's parents both carried the sickle cell trait, confirming his homozygous HbSS genotype.

**Table 1:** Lab Findings at Admission HR: heart rate; RR: respiratory rate; BP: blood pressure; FiO2: fraction of inspired oxygen; INR: international normalized ratio; PT: prothrombin time; Hb: hemoglobin; HCT: hematocrit; MCV: mean corpuscular volume; WBC: white blood cell count

Measurement	Value	Unit
Vitals Signs		
Temperature	98.20	°F
HR	77.00	bpm
RR	26.00	bpm
BP	134/79	mmHg
Sat	100.00	% O2
FiO2	4.00	L/min
Blood and Coagulation		
INR	1.55	
PT	16.00	s
Hb	6.90	g/dL
HCT	20.80	%
MCV	84.00	fL
Platelet	99000	/μL
WBC	8200	/μL
Neutrophil	56	%
Lymphocyte	21	%
Monocyte	5	%

**Figure 1:**
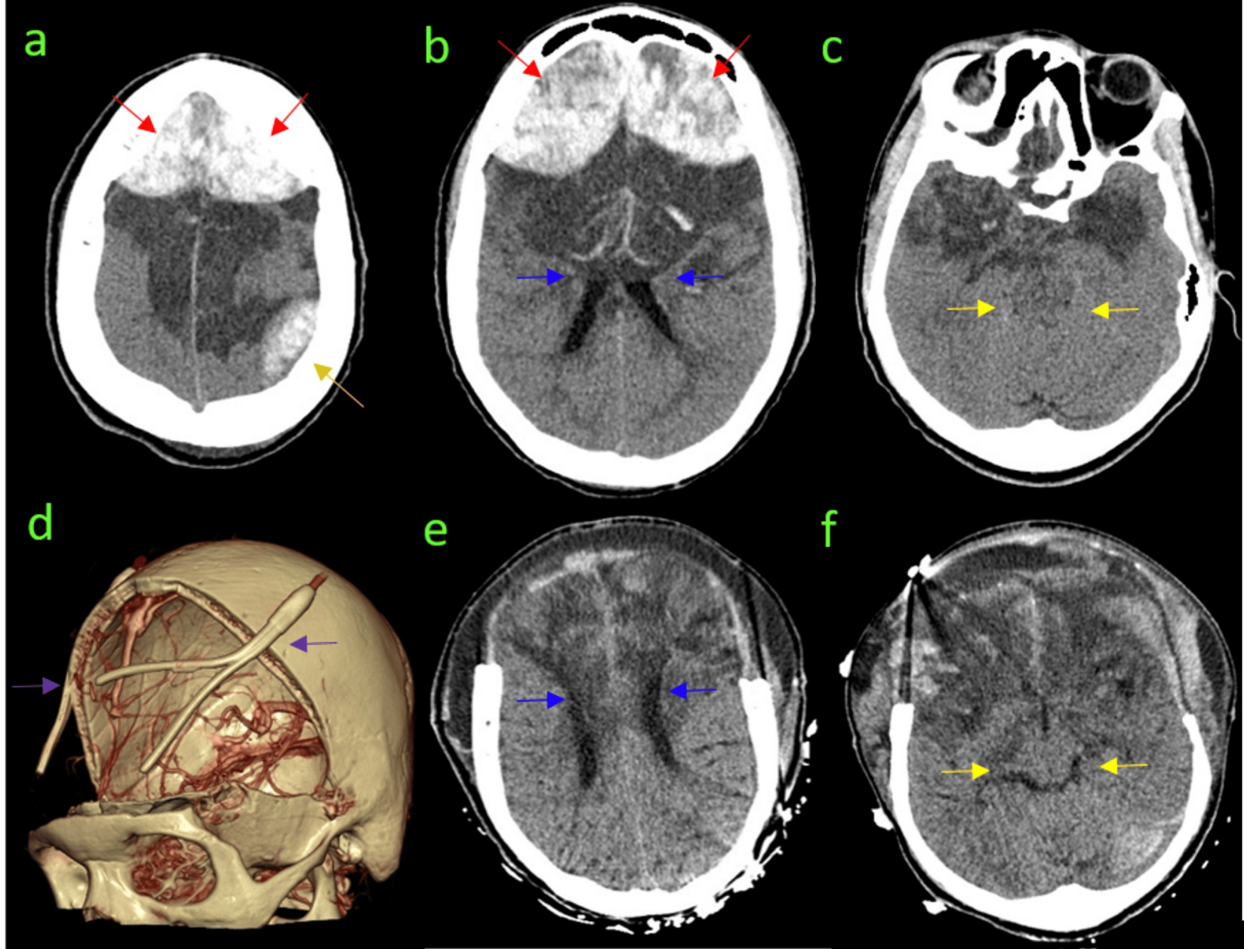
Admission and Post-operative CT (a-c) CT images depict a large bifrontal epidural hematoma (red arrows) and a left posterior parietal mixed-density epidural hematoma (orange arrow). There is a severe mass effect with the complete effacement of bilateral sulci and ventricular compression (blue arrows), indicating increased intracranial pressure. Entrapment and partial effacement of the basal cisterns (yellow arrows) are also noted. (d) A post-operative 3D reconstruction demonstrates the extensive craniotomy with bilateral Jackson-Pratt (JP) drains (purple arrows). (e, f) Post-operative CT axial image showing successful decompression with decreased effacement of skull base cisterns (yellow arrows) and ventricular system (blue arrows).

Upon arrival at our center, he repeated head CT with similar findings as described in transfer records: large bifrontal 8.3 x 5.5 x 9.1 cm with EDH with severe mass effect and a left minor posterior parietal EDH (Figures 1a-1c). Shortly after admission, in parallel with anemia and INR correction, the patient was taken to the operating room for a bifrontal bitemporal craniectomy with the evacuation of extradural hematomas and frontal sinus cranialization (Figure 1d). Two Jackson-Pratt (JP) drains were placed and the patient was in the ICU with strict blood pressure (BP) control.

**Figure 2:**
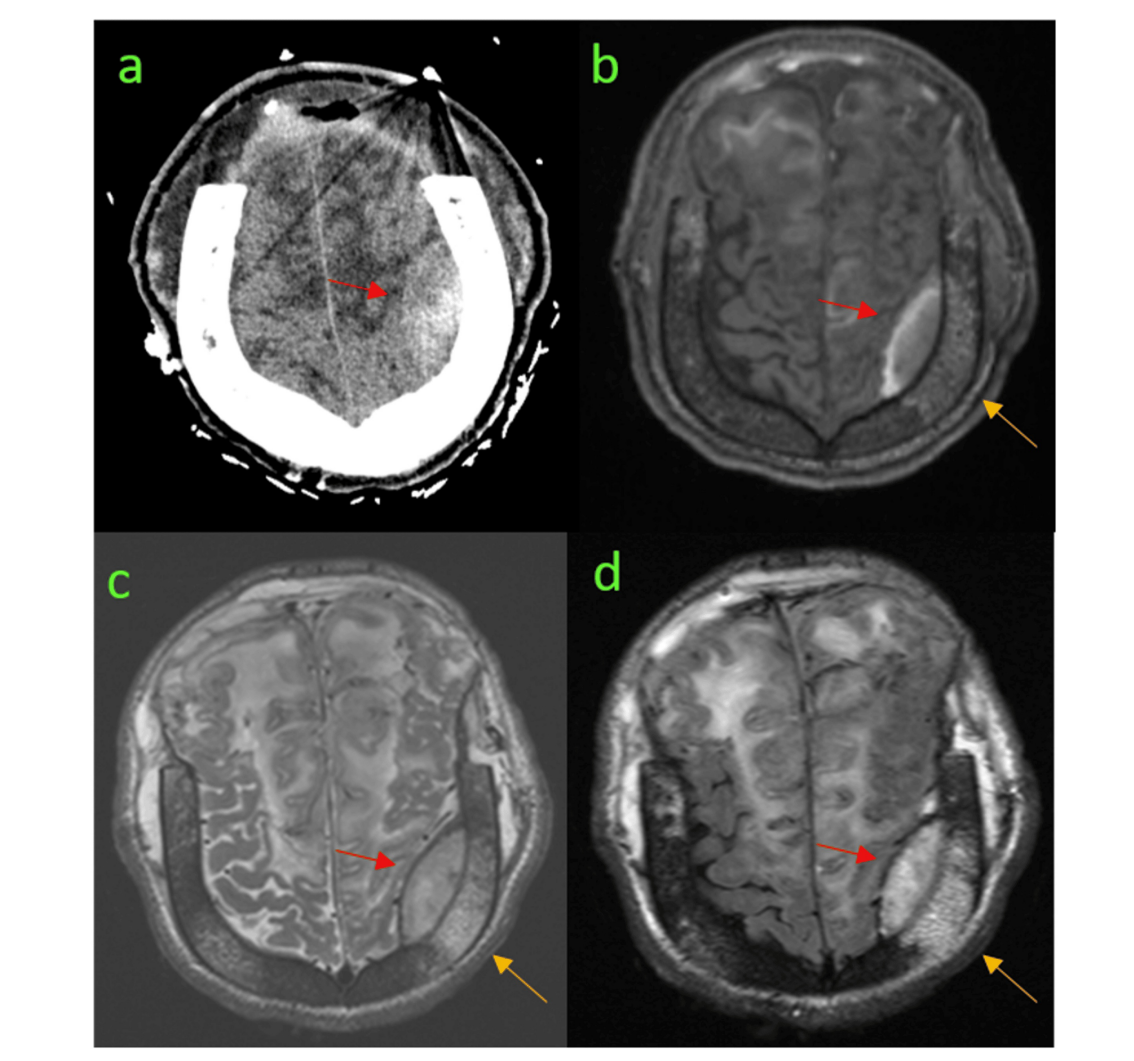
Bone Necrosis Over Epidural Hematoma (a) Post-operative CT scan showing epidural hematoma (EDH) (red arrow). (b-d) MRI images showing bone infarct (yellow arrow) above parietal EDH (red arrow) in T1 (b), T2 (c), and FLAIR (d). FLAIR: fluid-attenuated inversion recovery

Post-operative brain CT showed an overall improved mass effect with decreased effacement of skull base cisterns and ventricular system (Figures 1d-1e). Hemoglobin electrophoresis on post-operative day 1 was compatible with the past medical history of SCD (Table 2). Post-operative MRI demonstrated diffuse brain injury and hyperintensities on T1, T2, and fluid-attenuated inversion recovery (FLAIR) sequences surrounding the calvarium, suggestive of bone infarction (Figure 2).

**Table 2:** Hemoglobin Electrophoresis Post-operative Day 1 HbA: hemoglobin A; HbA2: hemoglobin A2; HbF: fetal hemoglobin

Hemoglobin Type	Percentage
HbA	79.4
HbA2	2.7
HbF	2.1
HbS/D/Q	15.8

The patient remained in the ICU for 22 days, during which BP and all other relevant parameters were carefully monitored and controlled. Five days after being transferred out of the ICU, he was discharged to a nursing facility. At the time of discharge, the neurologic examination showed an altered level of consciousness (eyes opening to stimulation), the ability to localize painful stimuli in both upper extremities, and preserved cough and gag reflexes. However, the pupils were fixed and dilated, with no response to light. The patient was bedridden and tracheotomized, and due to the severity of the injury, the prognosis remains poor, with a Rankin score of 5. Life-long rehabilitation and follow-up will be necessary, focusing on managing the sequelae of the patient’s condition.

## Discussion

SCD is an autosomal recessive disorder characterized by the presence of abnormal hemoglobin S, leading to various clinical complications such as vaso-occlusive crises, acute chest syndrome, and ischemic events [6]. Central nervous system complications are among the most severe and life-threatening outcomes of SCD [4]. While ischemic complications, such as stroke, are more commonly observed, hemorrhagic complications, including subdural, subarachnoid, parenchymal, and EDHs, can also occur, though they are much rarer. EDH, a collection of blood between the dura mater and the inner part of the skull, which is typically caused by trauma resulting in bleeding from the ruptured meningeal vessel, is exceedingly uncommon in the absence of a history of brain trauma [6], and its pathophysiology in SCD patients is not fully understood.

In patients with SCD, recurrent vaso-occlusive crises frequently result in hospitalizations. These crises are triggered by abnormal red blood cells leading to increased intramedullary pressure within the avascular bone marrow, causing pain and inflammation. In cases involving EDH, neurologic deterioration often follows a sickle cell crisis [8]. Several mechanisms have been proposed to explain the development of spontaneous EDH in SCD, including (1) bone infarction, which disrupts cortical bone and leads to epidural bleeding; (2) hyperproliferation of bone marrow that weakens the skull’s inner and outer margins, allowing for blood extravasation into the epidural space; and (3) rupture of fragile veins due to venous congestion from impaired drainage, possibly contributing to edema and hemorrhage [6].

We hypothesized that bone infarction was the primary contributing factor in this case. Although infarction of the frontal bone was not visible on the initial CT scan or subsequent imaging - since the bone flap had been removed to achieve brain decompression - we believe that the frontal bone was likely infarcted, given the clear infarction observed in the parietal bone overlying the left parietal EDH on the post-operative MRI.

In a recent review of 31 cases, Saha and Saha [9] found that 15 had evidence of bone infarction, 14 had no evidence, and two were unspecified. Notably, none of the bone infarctions were detected by CT scan: 13 were identified via MRI, one intra-operatively, and one through a bone scan. In contrast, among the 14 cases without bone infarction, only three underwent MRI. This suggests that bone infarction cannot be ruled out based solely on CT imaging, and highlights the importance of MRI for confirming bone infarction.

In cases of intracranial hemorrhages, such as EDH, combined with complex conditions like SCD, multidisciplinary collaboration is essential. For instance, the elevated INR observed in patients with SCD can be attributed to factors such as liver dysfunction and coagulation abnormalities [10]. This was a minor issue in our patient, as the Emergency Department was able to promptly correct the INR, allowing surgery to proceed safely. Post-operative care in the ICU is equally vital, particularly in controlling BP and managing fluids. Elevated BP can increase the risk of post-operative bleeding, while inadequate BP or fluid management can trigger vaso-occlusive crises [6], a common complication in sickle cell patients.

## Conclusions

This is a very rare case of bilateral spontaneous EDHs related to SCD, illustrating bone infarction as a possible mechanism. Our patient did not experience a prior sickling crisis before the onset of the EDH. Pre- and post-operative critical care, with emphasis on BP control, hydration, and vigilant laboratory monitoring, are essential. Although rare, hemorrhagic complications must be considered in SCD patients presenting with abrupt neurological symptoms, even in the absence of trauma or vaso-occlusive crises.
